# Resolutions of the Erie County Medical Society upon the Death of Dr. Gorham F. Pratt

**Published:** 1871-04

**Authors:** 


### Resolutions of the Erie County Medical Society upon the Death of Dr. Gorham F. Pratt.

At the meeting of the Erie County Medical Society, held on Saturday, the following preamble and resolutions were unanimously adopted:

*Whereas*, In the providence of God our associate, DR. GORHAM F. PRATT, has been removed by death from us, and

*Whereas*, Our late associate was esteemed as an honored member of this society; therefore be it

*Resolved*, That in the death of Dr. PRATT the society deplores the loss of its oldest and one of its most highly esteemed members, the profession of Buffalo and of Erie county the loss of a wise counsellor, the sick a sympathising and devoted friend, and this community a physician in whom implicit confidence was reposed in all times of need.

*Resolved*, That we deeply sympathize with the family of the deceased in their affliction and with this community in its irreparable loss.

*Resolved*, That we will ever cherish the memory of the departed, endeavor to emulate his many virtues, and profit by his wise example.

*Resolved*, That a copy of these resolutions be forwarded by the Secretary to the daily papers of the city, and Buffalo *Medical Journal* for publication, and an engrossed copy signed by the President and Secretary be transmitted to the family of the deceased.

C. C. F. GAY, JOHN HAUENSTEIN,
S. F. MIXER, P. H. STRONG,
C. C. WYCKOFF, *Committee.*

